# A TRPM4 Inhibitor 9-Phenanthrol Inhibits Glucose- and Glucagon-Like Peptide 1-Induced Insulin Secretion from Rat Islets of Langerhans

**DOI:** 10.1155/2017/5131785

**Published:** 2017-10-02

**Authors:** Zuheng Ma, Anneli Björklund, Md. Shahidul Islam

**Affiliations:** ^1^Department of Molecular Medicine and Surgery, Karolinska Institutet, Karolinska University Hospital, 171 76 Stockholm, Sweden; ^2^Department of Clinical Science and Education, Södersjukhuset, Karolinska Institutet, Research Center, 3rd Floor, 118 83 Stockholm, Sweden; ^3^Department of Emergency Care and Internal Medicine, Uppsala University Hospital, Uppsala University, Uppsala, Sweden

## Abstract

Pancreatic *β*-cells express several ion channels of the transient receptor potential family, which play important roles in mediating the stimulus-secretion coupling. One of these channels, the TRPM4 is a Ca^2+^-activated monovalent cation channel. This channel is inhibited by 9-phenanthrol, which also inhibits the TMEM16a Cl^−^ channel, and activates the Ca^2+^-activated K^+^ channel, K_ca_3.1. The net effects of ion-channel modulation by 9-phenantherol on the insulin secretion remain unclear. We tested the effects of 9-phenanthrol on glucose- and GLP-1-induced insulin secretion from isolated rat islets in static incubations. When applied to the islets in the presence of 3.3 mM glucose, 9-phenanthrol caused a small increase in insulin secretion (~7% of the insulin secretion stimulated by 10 mM glucose). 10 *μ*M 9-phenanthrol did not inhibit glucose- or GLP-1-induced insulin secretion. 20 *μ*M and 30 *μ*M 9-phenanthrol inhibited glucose-induced insulin secretion by ~80% and ~85%, respectively. Inhibition of the GLP-1-induced insulin secretion by 20 *μ*M and 30 *μ*M 9-phenanthrol was 65% and 94%, respectively. Our study shows that the major effect of 9-phenanthrol on the islets is a strong inhibition of insulin secretion, and we speculate that compounds related to 9-phenanthrol may be potentially useful in treating the pancreatogenous hyperinsulinemic hypoglycemia syndromes.

## 1. Introduction

Impairment of insulin secretion from the islets of Langerhans is one component of the mechanisms that underlie the pathogenesis of type 2 diabetes. The molecular mechanisms that lead to such impairments are not fully clear. Glucose, glucagon-like peptide-1 (GLP-1), and other insulin secretagogues stimulate secretion from the *β*-cells by involving a cascade of signaling events that are not fully understood. In this respect, depolarization of the plasma membrane potential, electrical activity, and Ca^2+^ signaling play pivotal roles in mediating the stimulus-secretion coupling in the *β*-cells.

Many ion channels, including the ATP-sensitive potassium (K_ATP_) channels, and the voltage-gated Ca^2+^ channels participate in mediating the periodic electrical activity and Ca^2+^ signaling in the *β*-cell [1]. There is evidence suggesting that some channels of the transient receptor potential (TRP) family mediate inward cationic currents for depolarizing the plasma membrane potential of the *β*-cells [2]. Marabita and Islam demonstrated that human *β*-cells express at least eight TRP channels, for example, TRPC1, TRPM4, TRPM7, TRPM2, TRPM3, TRPP2, TRPML1, and TRPML3 [3]. TRPM4 and TRPM5 are two closely related nonselective cation channels permeable to the monovalent cations, but almost impermeable to the divalent ones. These channels are activated by the intracellular free Ca^2+^ ion concentration ([Ca^2+^]_i_). Rat islets express both the TRPM4 and the TRPM5 channels. Krishnan et al. have shown that triphenylphosphine oxide, an inhibitor of the TRPM5 channel, inhibits insulin secretion from the rat islets [4]. Investigators have used 9-phenanthrol, a cell permeable hydroxytricyclic derivative, to test the effect of the inhibition of the TRPM4 channel on different physiological processes in many cells and tissues [5]. In contrast to the other inhibitors of TRPM4 channels, for example, glibenclamide and flufenamic acid, 9-phenanthrol is more selective for the TRPM4 channels. It inhibits the TRPM4 channels without inhibiting its closest relative, the TRPM5 channel, which is coexpressed with the TRPM4 channel in many cells [6].

Although 9-phenanthrol was initially discovered as an inhibitor of the TRPM4 channels, later studies showed that it also inhibits the transmembrane protein 16A (TMEM16A), a Ca^2+^-activated Cl^−^ channel [7]. In addition, it activates the Ca^2+^-activated K^+^ channel (K_ca_3.1) [8]. All these actions of 9-phenanthrol on these three ion channels of the *β*-cells are expected to inhibit insulin secretion.

The effects of 9-phenanthrol on the insulin secretion from the rat islets have not been carefully studied. In this study, we demonstrate that 9-phenanthrol inhibits glucose- and GLP-1-induced insulin secretion from the isolated rat islets.

## 2. Materials and Methods

### 2.1. Chemicals

RPMI-1640 medium and other cell culture materials were purchased from Life Technologies, Stockholm, Sweden. Collagenase, 9-phenanthrol, and fetal bovine serum (FBS) were from Sigma-Aldrich, Stockholm, Sweden. Glucagon-like peptide 1 (7–37) was from Bachem, Switzerland.

### 2.2. Preparation of Islets from Rats

Ethical approval for the study was obtained from the Northern Stockholm ethical committee on experimental animal care. All the experiments were done following the guidelines of the Swedish National Board for Laboratory Animals. Male Sprague-Dawley rats were from Scanbur AB (Sollentuna, Sweden). We followed the ethical guidelines of the Karolinska Institute for the care and use of laboratory animals. The rats were kept in a 12-hour (6 a.m. to 6 p.m.) light/dark cycle with free access to standard diet and water. At the time of the experiments, the rats weighed 250 to 350 g.

The animals were humanely killed by inhalation of CO_2_. Collagenase (0.1% wt/vol) was injected into the pancreatic duct. The pancreas was digested by collagenase in Hank's balanced salt solution. Islets were separated by centrifugation and density gradient separation with Histopaque 1077 and Histopaque 1119. The islets were handpicked by observing them under a stereomicroscope and kept in petri dishes containing RPMI-1640 medium, glucose (11 mM), glutamine (2 mM), FBS (10% vol/vol), benzylpenicillin (100 U/mL), and streptomycin (100 *μ*g/mL) [4]. The islets were then cultured for 24 hours in an incubator at 37°C in 5% CO_2_. Later on, the islets were transferred to petri dishes containing 5 mL of Krebs-Ringer bicarbonate buffer, HEPES (10 mM), BSA (0.2%), and glucose (3.3 mM). On the day of the experiments, the islets were preincubated in this solution for 30 minutes at 37°C in 5% CO_2_.

### 2.3. Measurement of Insulin Secretion from the Islets

We chose islets that were of similar sizes on visual inspection, for all the experiments. The islets were incubated in groups of 3 in 300 *μ*L of Krebs-Ringer bicarbonate buffer containing glucose (3.3, 10, or 16.7 mM), with or without other pharmacological agents, for 1 hour at 37°C in a water bath under continuous shaking. After 1 hour, the supernatant was carefully removed and stored at −20°C [4].

In separate experiments, we also measured the insulin contents of the islets and expressed the released insulin as percentage of the insulin content. The insulin content was measured after sonication of the islets for 10 to 15 seconds, followed by extraction of insulin overnight at 4°C in 200 *μ*L of acid-ethanol (70% vol/vol).

Immunoreactive insulin was measured by radioimmunoassay using rat insulin as standard and insulin antibodies (Fitzgerald, United States).

### 2.4. Statistical Analysis

The results were expressed as mean, standard deviation (SD), and the 95% confidence interval (CI). One-way analysis of variance (ANOVA) with Sidak-Holm post hoc test was used for testing the statistical significance. Data were analyzed by means of the SigmaPlot package (Systat Software Inc., San Jose, CA, USA).

## 3. Results

**Figure 1:**
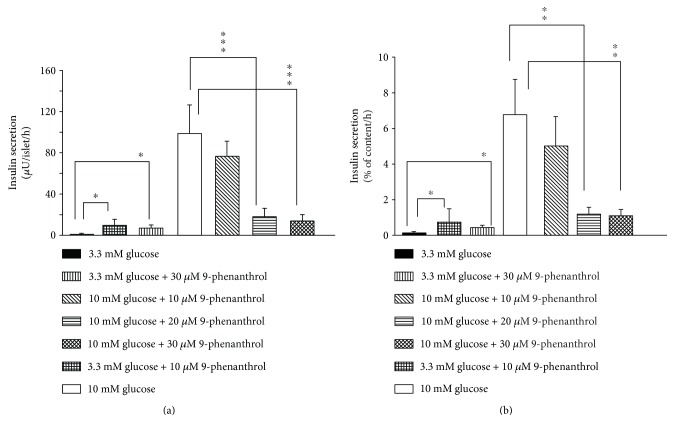
Effect of 9-phenanthrol on glucose-stimulated insulin secretion from isolated rat islets of Langerhans. Insulin secretion was studied in batch incubations where groups of three islets were incubated for 1 hour in 3.3 mM or 10 mM glucose in the presence of different concentrations of 9-phenanthrol, as indicated at the bottom of the figure. In (a), insulin secretion is expressed as *μ*U/islet/hour and in (b), insulin secretion is expressed as percent of total insulin content per hour. ^∗^*P* ≤ 0.05, ^∗∗^*P* ≤ 0.01, and ^∗∗∗^*P* ≤ 0.001 (one-way ANOVA).

Insulin secretion by 3.3 mM glucose was increased by 10 *μ*M 9-phenanthrol (Figure 1(a)); mean insulin secretion (*μ*U/islet/hour) by 3.3 mM glucose was 1.78 (SD 0.75, 95% CI 1.15–2.41, *n* = 8), and that in the presence of 10 *μ*M 9-phenanthrol was 9.42 (SD 5.88, 95% CI 0.05–18.79, *n* = 4, *P* < 0.05). Thirty micromolar 9-phenanthrol also induced similar increase of basal insulin secretion (mean 6.75, SD 3.5, 95% CI 3.23–10.27, *n* = 4, *P* ≤ 0.05). However, the magnitude of the increased insulin secretion by 9-phenanthrol was small (only ~7% of the insulin secretion stimulated by 10 mM glucose).

To test whether the stimulatory effect of 9-phenanthrol on insulin secretion at low glucose was due to depolarization of the *β*-cells, we used diazoxide, which hyperpolarizes *β*-cells by activating the K_ATP_ channel, and found that diazoxide did not inhibit this effect of 9-phenanthrol. Mean insulin secretion (*μ*U/islet/hour) in the presence of 3.3 mM glucose was 0.72 (SD 0.20, 95% CI 0.42–1.04, *n* = 4). Mean insulin secretion in the presence of 3.3 mM glucose plus 30 *μ*M 9-phenanthrol was 1.95 (SD 0.79, 95% CI 0.69–3.21, *n* = 4), and that in the presence of 3.3 mM glucose, 30 *μ*M 9-phenanthrol, and 100 *μ*M diazoxide was 1.82 (SD 0.93, 95% CI 0.34–3.30, *n* = 4, *P* NS).

Stimulation of the islets with 10 mM glucose caused a 55-fold increase in insulin secretion compared to the secretion by 3.3 mM glucose (*P* ≤ 0.001) (Figure 1(a)) indicating that the islets were functionally intact. Insulin secretion stimulated by 10 mM glucose was not significantly inhibited by 10 *μ*M 9-phenanthrol but was significantly inhibited by 20 *μ*M and 30 *μ*M 9-phenanthrol. Mean insulin secretion (*μ*U/islet/hour) by 10 mM glucose was 98.75 (SD 27.69, 95% CI 75.60–121.90, *n* = 8), and that in the presence of 10 *μ*M 9-phenanthrol was 76.50 (SD 14.47, 95% CI 53.47–99.53, *n* = 4, *P* NS). Mean insulin secretion by 10 mM glucose in the presence of 20 *μ*M 9-phenanthrol was 18.25 (SD 7.63, 95% CI 6.11–30.39, *n* = 4, *P* ≤ 0.001), and that in the presence of 30 *μ*M 9-phenanthrol was 13.25 (SD 6.89, 95% CI 2.28–24.22, *n* = 4, *P* ≤ 0.001). Inhibition of insulin secretion by 20 *μ*M 9-phenanthrol was not significantly different from that by 30 *μ*M 9-phenanthrol.

We also tested the effect of 9-phenanthrol on insulin secretion stimulated by 16.7 mM glucose. Consistent with the results described above, 10 *μ*M 9-phenanthrol did not, whereas 30 *μ*M 9-phenanthrol did inhibit insulin secretion by 16.7 mM glucose (61% inhibition, *P* ≤ 0.001). Mean insulin secretion (*μ*U/islet/hour) by 16.7 mM glucose was 71.33 (SD 12.85, 95% CI 39.46–103.2, *n* = 4); that in the presence of 10 *μ*M 9-phenanthrol was 50.66 (SD 9.07, 95% CI 28.17–73.15, *n* = 4); and that in the presence of 30 *μ*M 9-phenanthrol was 27.33 (SD 13.05, 95% CI −5.02–59.68, *n* = 4). Inhibition of secretion by higher concentrations of 9-phenanthrol, namely, 60 *μ*M and 100 *μ*M, was not significantly different from that by 30 *μ*M 9-phenanthrol.

To control for the variability of the size and the insulin content of the islets, we calculated insulin secretion as percentage of the total insulin content of the islets (Figure 1(b)). Consistent with the results presented above, insulin secretion by 10 mM glucose was not significantly inhibited by 10 *μ*M 9-phenanthrol but was significantly inhibited by 20 *μ*M 9-phenanthrol (*P* ≤ 0.01) and 30 *μ*M 9-phenanthrol (*P* ≤ 0.01). In the presence of 3.3 mM glucose, 10 *μ*M and 30 *μ*M 9-phenanthrol increased insulin secretion (*P* ≤ 0.05).

**Figure 2:**
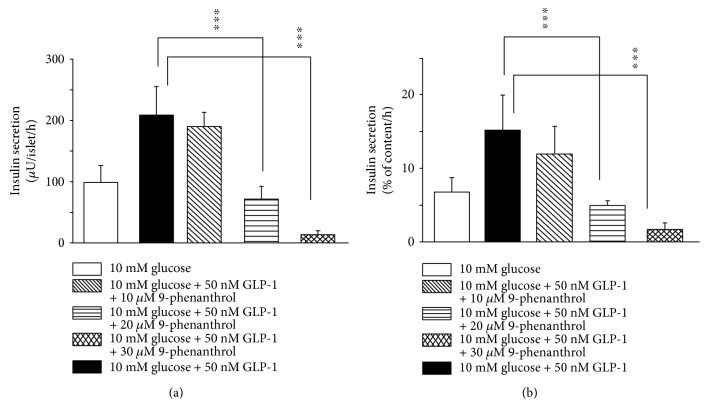
Effect of 9-phenanthrol on GLP-1-induced insulin secretion from isolated rat islets of Langerhans. Insulin secretion was studied in batch incubations where groups of three islets were incubated for 1 hour in 10 mM glucose or 10 mM glucose +50 nM GLP-1, in the presence of different concentrations of 9-phenanthrol, as indicated at the bottom of the figure. In (a), insulin secretion is expressed as *μ*U/islet/hour and in (b), insulin secretion is expressed as percent of total insulin content per hour. ^∗∗∗^*P* ≤ 0.001 (one-way ANOVA).

Figure 2 shows the effect of 9-phenanthrol on insulin secretion stimulated by 10 mM glucose plus 50 nM glucagon-like peptide 1 (GLP-1). GLP-1 increased glucose-induced insulin secretion by 110%. Mean insulin secretion (*μ*U/islet/hour) by 10 mM glucose plus 50 nM GLP-1 was 208.12 (SD 47.40, 95% CI 168.49–247.65, *n* = 8), and that in the presence of 10 *μ*M 9-phenanthrol was 190.00 (SD 23.33, 95% CI 152.85–227.12, *n* = 4, *P* NS). Mean insulin secretion (*μ*U/islet/hour) by glucose plus GLP-1 in the presence of 20 *μ*M 9-phenanthrol was 71.75 (SD 20.67, 95% CI 38.86–104. 64, *n* = 4, *P* ≤ 0.001), and that in the presence of 30 *μ*M of the inhibitor was 13.25 (SD 6.89, 95% CI 2.28–24.22, *n* = 4, *P* ≤ 0.001). Inhibition of GLP-1-induced insulin secretion by 20 *μ*M 9-phenanthrol was 65% whereas that by 30 *μ*M 9-phenanthrol was 94% (*P* ≤ 0.001). In another set of experiments, we tested the effect of 9-phenanthrol on insulin secretion when *β*-cells were maximally stimulated by 16.7 mM glucose plus 50 nM GLP-1 and found that 9-phenanthrol (30 *μ*M) inhibited maximal insulin secretion by 88%. Insulin secretion (*μ*U/islet/hour) in the presence of 16.7 mM glucose plus 50 nM GLP-1 was 325 (SD 73, 95% CI 209–441), and that in the presence of 16.7 mM glucose, 50 nM GLP-1 plus 30 *μ*M 9-phenanthrol was 36.75 (SD 15.92, 95% CI 11.42–62.08, *n* = 4, *P* ≤ 0.001).

To test if the difference in insulin secretion could be due to the variability of the size or the insulin content of the islets, we measured insulin content of the islets and expressed insulin secretion as percentage of the insulin content (Figure 2(b)). When expressed this way, we found that 10 *μ*M 9-phenanthrol did not significantly inhibit insulin secretion, whereas 20 *μ*M and 30 *μ*M 9-phenanthrol inhibited insulin secretion significantly (*P* ≤ 0.001).

To test the effect of the longer term exposure of the islets to 9-phenanthrol, we cultured the islets in the presence of 10 *μ*M 9-phenanthrol for 24 hours. This treatment did not significantly alter the insulin secretion in response to 16.7 mM glucose or the insulin content of the islets compared to the control islets that were cultured without 9-phenanthrol. Mean insulin secretion (*μ*U/islet/hour) in response to 16.7 mM glucose in the control islets was 1180.75 (SD 213.91, 95% CI 840.44–1521.06, *n* = 4), and that in the islets treated with 10 *μ*M 9-phenanthrol for 24 hours was 1342 (SD 167.00, 95% CI 1076.32–1607.68, *n* = 4, *P* NS). However, when we cultured the islets in 30 *μ*M 9-phenanthrol, for 24 hours, the islets appeared disintegrated on inspection under the microscope.

## 4. Discussion

The main finding of this study was that 9-phenanthrol inhibited glucose- and GLP-1-induced insulin secretion from the isolated rat islets in static incubations. When used at a concentration of 10 *μ*M, 9-phenanthrol did not inhibit glucose- or GLP-1-induced insulin secretion significantly, but 20 *μ*M and 30 *μ*M 9-phenanthrol inhibited glucose-induced insulin secretion by ~80% and ~85%, respectively. Inhibition of the GLP-1-induced insulin secretion by 20 *μ*M and 30 *μ*M 9-phenanthrol was 65% and 94%, respectively. While the glucose-induced insulin secretion was maximally inhibited by 20 *μ*M 9-phenanthrol, a higher concentration, that is, 30 *μ*M was needed for maximal inhibition of the GLP-1-induced insulin secretion. The fact that 20–30 *μ*M 9-phenanthrol inhibited insulin secretion is consistent with the fact that the inhibition of insulin secretion was due to the inhibition of the TRPM4 channels because the IC_50_ of 9-phenanthrol for the transfected or the endogenous TRPM4 channels has been shown to be 20–30 *μ*M in many cells [5, 6]. The degree of inhibition of insulin secretion by 20–30 *μ*M 9-phenanthrol is also consistent with the degree of inhibition of the activity of the TRPM4 channels by the similar concentrations of the substance reported in many cell types [5]. Glucose stimulation depolarizes the plasma membrane potential and increases the [Ca^2+^]_i_ in the *β*-cells; both of these events increase the activity of the TRPM4 channels. The inhibition of insulin secretion from rat islets by 9-phenanthrol is consistent with the roles of the TRPM4 channel in mediating electrical activity and insulin secretion that has been demonstrated in several rodent insulinoma cell lines [9]. More recently, Shigeto et al., by using a variety of methods including the use of 9-phenanthrol, have demonstrated that TRPM4 is involved in the GLP-1-induced insulin secretion from mouse islets [10].

Crutzen et al. have recently demonstrated that chloride efflux through a Ca^2+^-activated Cl^−^ channel, anoctamine 1 (Ano1), also called “transmembrane member 16A” (TMEM16A), is involved in mediating the glucose-stimulated insulin secretion [11]. As mentioned earlier, this channel is also inhibited by 9-phenanthrol at similar concentrations that inhibit the TRPM4 channel [7]. Activation of the Ca^2+^-activated K^+^ channel (K_ca_3.1) of the *β*-cells causes hyperpolarization of the plasma membrane potential and inhibits insulin secretion [12]. This channel is activated by 9-phenanthrol in the similar concentrations that inhibit the TRPM4 channel [8]. It is thus likely that the strong inhibition of insulin secretion by 9-phenanthrol in our study is the net result of the inhibition of the TRPM4 and the TMEM16A channels and the activation of the K_ca_3.1 channel.

We found that the effect of 9-phenanthrol on insulin secretion was glucose dependent. While it inhibited insulin secretion triggered by 10 or 16.7 mM glucose with or without GLP-1, it actually stimulated insulin secretion when applied to the islets in the presence of 3.3 mM glucose. The magnitude of this stimulation was however only 7% of the insulin secretion stimulated by 10 mM glucose. The mechanism of the stimulation of insulin secretion by 9-phenanthrol at low glucose is unclear. Our experiments with diazoxide suggest that this was not due to depolarization of the *β*-cells. It is also unlikely to be due to nonspecific toxic effect of the substance on the *β*-cells. In general, the substance shows low cellular toxicity [6]. It has been shown that the effect of 9-phenanthrol on the electrical activity of the cardiac muscle cells is reversible, further suggesting lack of any major toxic effects of the substance when used for experiments over short time [13]. We speculate that the stimulation of insulin secretion at 3.3 mM glucose could be due to the effect of 9-phenanthrol on the exocytotic machinery of the *β*-cell.

## 5. Conclusions

We have demonstrated that 9-phenanthrol, an inhibitor of the TRPM4 and the TMEM16A channels and an activator of the K_ca_3.1 channel, strongly inhibits both the glucose- and the GLP-1-induced insulin secretion. We speculate that compounds related to 9-phenanthrol could potentially be useful, for instance, in the development of drugs for the treatment of patients with pancreatogenous hyperinsulinemic hypoglycemia syndromes [14].
